# Supplementary material for: Structured expert judgement approach of the health impact of various chemicals and classes of chemicals
Source: PLoS One. 2024 Jun 24;19(6):e0298504. doi: 10.1371/journal.pone.0298504 (PMC11195936; doi:10.1371/journal.pone.0298504)
Supplement: S1 Fig — (PDF) [file pone.0298504.s001.pdf]

```
                       Range graph of input data
Item no.:   1 Item name: CAL01 Scale: uni
Experts
  1                   [------------------*-------------------------------------]
  2    [--------------*------------------]
  3 |
  4 [--*---]
  5 [--*---]
  6                                  [---*------]
  7                                          [-------------------*------------]
  8        [------*--------------]
  9    [----*---------]
PW10   [=============*========================]
EW  [===========*==========================================================]
Real::::::::::::::::::::::::::::::::#:::::::::::::::::::::::::::::::::::::::::::
                                   838
     5                                                                      2000

Item no.:   2 Item name: CAL02 Scale: uni
Experts
  1 |
  2 [--------*---------------------------------------------]
  3 |
  4 [----*------------]
  5    [----------*-----------------------------------------------------------]
  6  [---*---]
  7 |
  8 |
  9  [-------*--------]
PW1 [*=================================================]
EW  [=*==============================================]
Real#:::::::::::::::::::::::::::::::::::::::::::::::::::::::::::::::::::::::::::
     28
     2                                                                     4E004

Item no.:   3 Item name: CAL03 Scale: uni
Experts
  1     [-------------------*------------------------------------------------]
  2 |
  3 |
  4 [*--]
  5 |
  6   [-*----]
  7   [*-]
  8     [-------------------*------------------------]
  9 [-*-]
PW1 *=]
EW  [=*==============================================]
Real::::::::::::::#:::::::::::::::::::::::::::::::::::::::::::::::::::::::::::::
                6.369E005
    50                                                                     3E006

Item no.:   4 Item name: CAL04 Scale: uni
Experts
  1 *]
  2 [----------------------------*--------------------------------------------]
  3 |
  4 [*---]
  5 |
  6 |
  7 |
  8 *]
  9  [*]
PW1 *=========================================================]
EW  *================================================]
Real:#::::::::::::::::::::::::::::::::::::::::::::::::::::::::::::::::::::::::::
     8.404E004
    10                                                                     5E006

Item no.:   5 Item name: CAL05 Scale: uni
Experts
  1 |
  2 |
  3 |
  4                   [----------------------------*---------------------------]
  5 |
  6                   [----*--------]
  7 |
  8 |
  9                             [--------*-----------------]
PW1 |
EW  *===========================================================]
Real#:::::::::::::::::::::::::::::::::::::::::::::::::::::::::::::::::::::::::::
     0.262
    0.1                                                                       80

Item no.:   6 Item name: CAL06 Scale: uni
Experts
  1 |
  2 |
  3 |
  4             [------------------------*------------------------------------]
  5 *]
  6      [*-]
  7 |
  8 |
  9             [------------------------*------------------------------------]
PW1 |
EW  *===============================================================]
Real#:::::::::::::::::::::::::::::::::::::::::::::::::::::::::::::::::::::::::::
     0.041
    0.02                                                                      30

Item no.:   7 Item name: CAL07 Scale: uni
Experts
  1 |
  2 |
  3 |
  4       [------------------------*-----------------]
  5 |
  6    *]
  7 |
  8 |
  9                          [-------------------------*------------------------]
PW1 |
EW  *============================================================]
Real#:::::::::::::::::::::::::::::::::::::::::::::::::::::::::::::::::::::::::::
     0.05
    0.01                                                                      60

Item no.:   8 Item name: CAL08 Scale: uni
Experts
  1 [*-]
  2          [--------*---------------------------------------------------------]
  3 [---------------------*-----]
  4 |
  5      [---*-]
  6  [-*-]
  7   [*---------]
  8          [----*-]
  9 [*]
PW10 [============*===========================================================]
EW  [===*==========================================]
Real:::::::::::::::::::::::::::::::::#::::::::::::::::::::::::::::::::::::::::::
                                 1.68E005
    20                                                                     4E005

Item no.:   9 Item name: CAL09 Scale: uni
Experts
  1              [-------*-------]
  2 [------------------------------------*-------------------------------------]
  3  [*]
  4 [---*----]
  5              [-------*-------]
  6              [--*-----------]
  7              [---*------------------]
  8   [----*-]
  9          [---------*----------]
PW10   [===============*==========================================]
EW   [=============*========================================]
Real:::::#::::::::::::::::::::::::::::::::::::::::::::::::::::::::::::::::::::::
          101
    20                                                                      1000



Item no.:  10 Item name: CAL10 Scale: uni
Experts
  1   [-*----]
  2 *--]
  3 [-*-]
  4     [------------------*-----------------------------------------------------]
  5 *]
  6 *]
  7 [-*-]
  8 [---*---------]
  9       [----------------*--------------]
PW1 *=======]
EW  [=*==============================================]
Real#:::::::::::::::::::::::::::::::::::::::::::::::::::::::::::::::::::::::::::
     1800
    750                                                                  1.5E005

Item no.:  11 Item name: CAL11 Scale: uni
Experts
  1  [-------*------]
  2 [----*-----------------]
  3                       [-------*------]
  4  [-------*------]
  5            [---*------]
  6        [*--]
  7                       [------------------------------------*-------------]
  8  [-------*------]
  9          [--*-------]
PW1 [=====*================]
EW   [==========*==================================================]
Real:::::::::::#::::::::::::::::::::::::::::::::::::::::::::::::::::::::::::::::
               346
    175                                                                     1200

Item no.:  12 Item name: CAL12 Scale: uni
Experts
  1   [*---]
  2 [---*-]
  3      [-*---]
  4   [--*-]
  5      [-*---]
  6   [---*----]
  7            [----------------------*-------------------------------------]
  8   [---*--]
  9    [---*-------]
PW1 [===*====]
EW   [=====*=============================================]
Real::::::::::::::::::::#:::::::::::::::::::::::::::::::::::::::::::::::::::::::
                        31.54
     4                                                                       100

Item no.:  13 Item name: CAL13 Scale: uni
Experts
  1      [---*]
  2      [----------------*----------------------------------------------------]
  3      [---*--]
  4          [---------*------------------------------------------------------]
  5             [---*-----]
  6      [-----------*---------------------]
  7        [--*-]
  8 [*-]
  9          [----*-------]
PW10 [=========*===============================================================]
EW  [==========*=================================================]
Real::::#:::::::::::::::::::::::::::::::::::::::::::::::::::::::::::::::::::::::
       42.58
    30                                                                       250

Item no.:  14 Item name: CAL14 Scale: uni
Experts
  1 [-----*]
  2   [------------------------*--------------------------------------------]
  3            [-----*--]
  4            [------------------*---------------------------]
  5      [---*----]
  6         [--------------*------------------]
  7       [-*----]
  8    [-*-----]
  9            [---------------*--------------]
PW1 [======*==============================================================]
EW    [==========*=============================================]
Real::::::#:::::::::::::::::::::::::::::::::::::::::::::::::::::::::::::::::::::
          83.2
    60                                                                       300

Item no.:  15 Item name: CAL15 Scale: uni
Experts
  1                  [-------------------------------------*-----------------]
  2 |
  3 |
  4 |
  5 |
  6 |
  7 |
  8 |
  9 |
PW1 *===]
EW  *=============================================================]
Real#:::::::::::::::::::::::::::::::::::::::::::::::::::::::::::::::::::::::::::
     1.44E004
    2000                                                                   2E007

Item no.:  16 Item name: CAL16 Scale: uni
Experts
  1 |
  2 *]
  3 *]
  4               [-----------------------------*----------------------------]
  5 []
  6 [-----*--------]
  7 *]
  8 *]
  9               [--------------*--------------]
PW1 *]
EW  [*=======================================================]
Real#:::::::::::::::::::::::::::::::::::::::::::::::::::::::::::::::::::::::::::
     -0.174
    -0.174                                                                    25

Item no.:  17 Item name: CAL17 Scale: uni
Experts
  1 |
  2 |
  3 |
  4                                                  [-----------------------*---]
  5 |
  6                                                       [-------------*----]
  7 |
  8 |
  9                                                  [----*----]
PW1 |
EW  *===================================================================]
Real#:::::::::::::::::::::::::::::::::::::::::::::::::::::::::::::::::::::::::::
     0.61
    0.3                                                                       80
```
