# Supplementary figures and images for: Structured expert judgement approach of the health impact of various chemicals and classes of chemicals
Source: PLoS One. 2024 Jun 24;19(6):e0298504. doi: 10.1371/journal.pone.0298504 (PMC11195936; doi:10.1371/journal.pone.0298504)

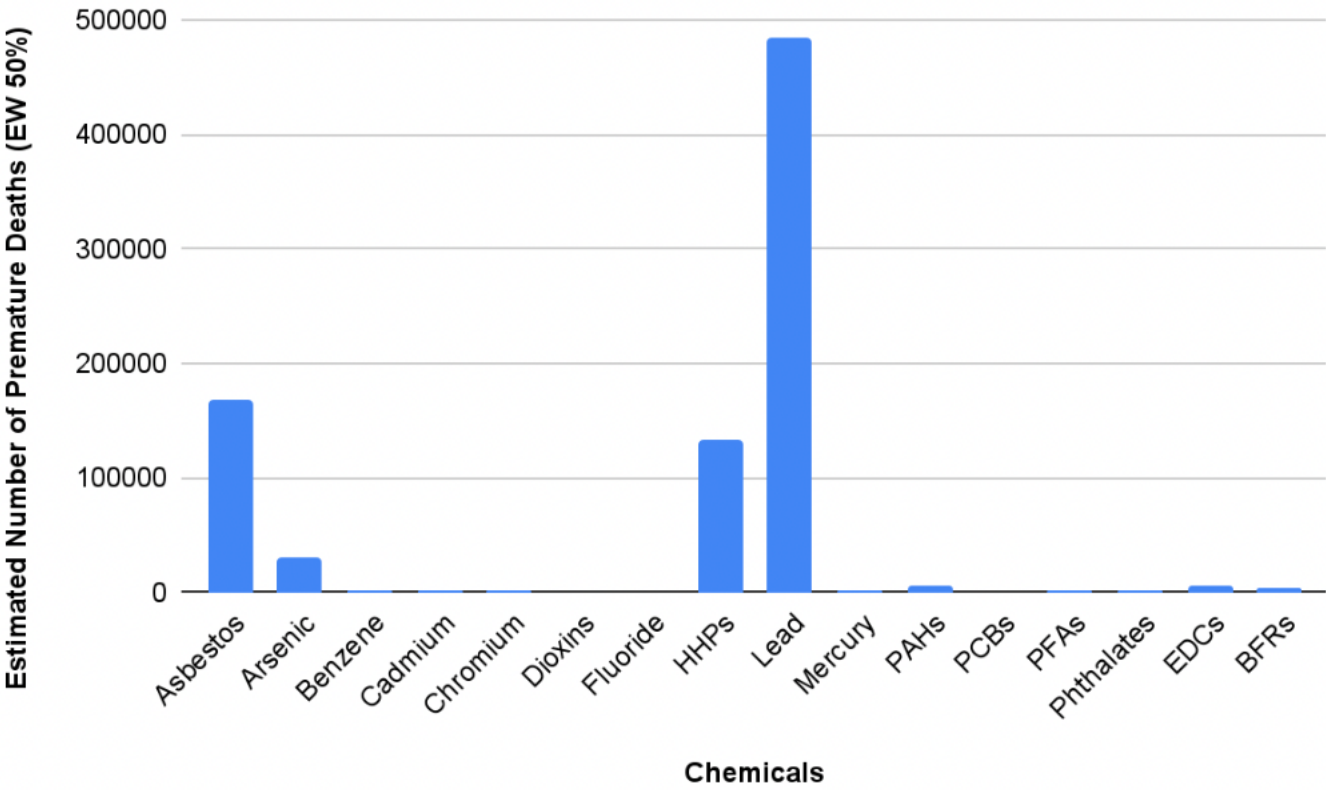

Supplement: S2 Fig — (PDF) [file pone.0298504.s002.pdf]

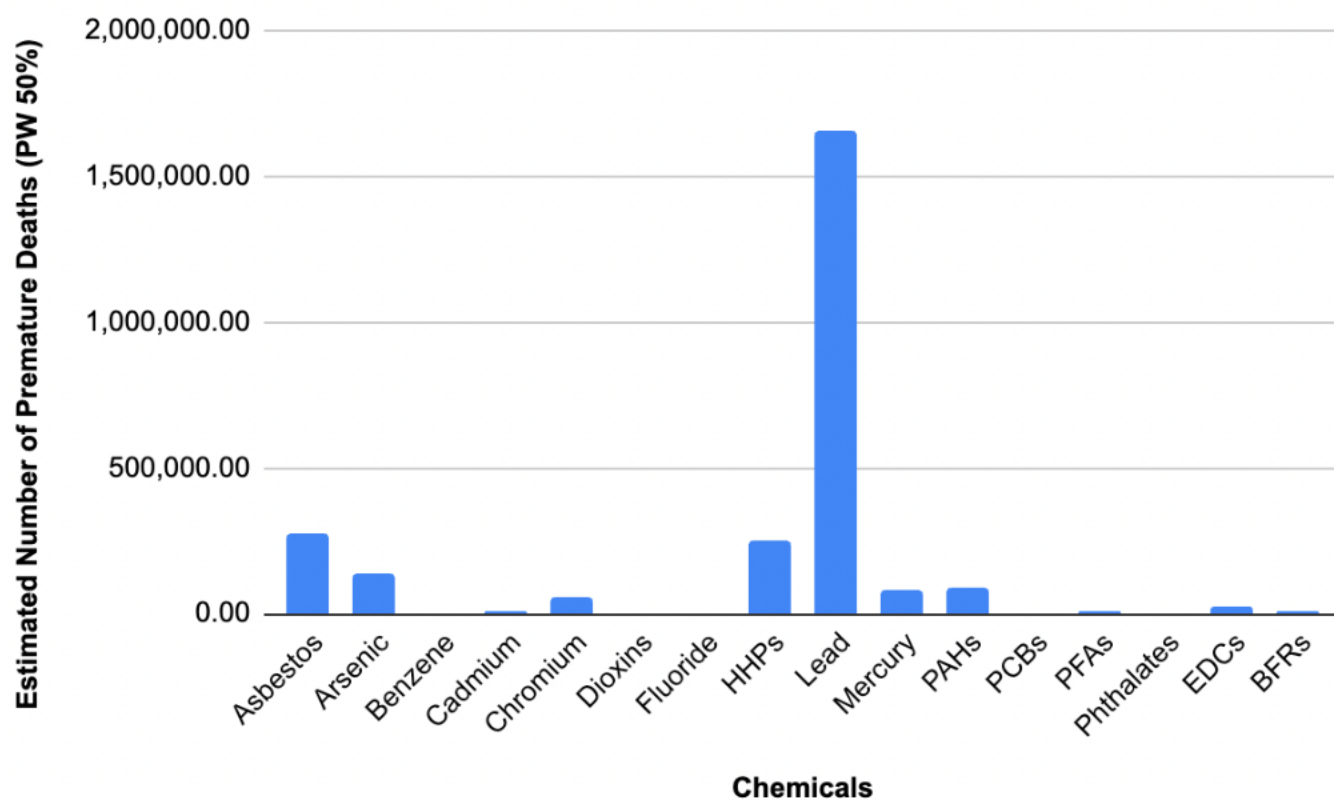

Supplement: S3 Fig — (PDF) [file pone.0298504.s003.pdf]
