# Supplementary material for: Structured expert judgement approach of the health impact of various chemicals and classes of chemicals
Source: PLoS One. 2024 Jun 24;19(6):e0298504. doi: 10.1371/journal.pone.0298504 (PMC11195936; doi:10.1371/journal.pone.0298504)
Supplement: S1 Table — (DOCX) [file pone.0298504.s004.docx]

**S1 Table: Summary of Experts and their Affiliations**

| **Expert Name** | **Expert Affiliation** |
| --- | --- |
| Dr. Roberto Bertollini | Member of the Scientific Committee on Health, Environment and Emerging Risks (SCHEER)  European Commission DG Sante |
| Dr. Patrick Breysse | Johns Hopkins’ Bloomberg School of Public Health |
| Dr. Jack Caravanos | New York University’s School of Global Public Health |
| Dr. Lillian Corra | Global Alliance on Health and Pollution |
| Dr. David Hanrahan | Pure Earth |
| Dr. Howard Hu | University of Southern California’s Keck School of Medicine |
| Dr. Amalia Laborde | Republic University of Montevideo, Uruguay |
| Dr. Ernesto Sánchez Triana | World Bank |
| Dr. Mona Wells | The Meadows Center for Water and the Environment |
