# Supplementary material for: Structured expert judgement approach of the health impact of various chemicals and classes of chemicals
Source: PLoS One. 2024 Jun 24;19(6):e0298504. doi: 10.1371/journal.pone.0298504 (PMC11195936; doi:10.1371/journal.pone.0298504)
Supplement: S2 Table — (DOCX) [file pone.0298504.s005.docx]

**S2 Table: Elicited Metals and their Abbreviations**

| **Chemical** | **Abbreviation** |
| --- | --- |
| Asbestos | ASB |
| Arsenic | AS |
| Benzene | BZ |
| Cadmium | CD |
| Chromium | CR |
| Dioxins | DF |
| Fluoride | FF |
| Highly Hazardous Pesticides | HHPs |
| Lead | PB |
| Mercury | HG |
| Polycyclic Aromatic Hydrocarbons | PAHs |
| Polychlorinated Biphenyls | PCBs |
| Polyfluorinated Substances | PFAs |
| Phthalates | FH |
| Endocrine Disrupting Chemicals | EDCs |
