# Supplementary material for: Structured expert judgement approach of the health impact of various chemicals and classes of chemicals
Source: PLoS One. 2024 Jun 24;19(6):e0298504. doi: 10.1371/journal.pone.0298504 (PMC11195936; doi:10.1371/journal.pone.0298504)
Supplement: S3 Table — (DOCX) [file pone.0298504.s006.docx]

**S3 Table: Summary of HICs and LMICs most impacted by chemical exposures, and their associated exposure pathways**

| **Chemical** | **High Income Countries Most Impacted** | **Low and Middle Income Countries Most Impacted** | **Most Severe Major Exposure Pathways** |
| --- | --- | --- | --- |
| **Asbestos** | U.S.  Canada  Australia  U.K. | Brazil  China  India  Russia | Occupational (mining)  Environmental Airborne |
| **Arsenic** | U.S.  Australia  HICs located within the Andes (e.g. Chile, Argentina) | Bangladesh  China  India  Southeast Asia (e.g. Vietnam, Cambodia) | Drinking/Groundwater  Occupational |
| **Benzene** | U.S.  OPEC Members | China  India Venezuela  OPEC Members | Occupational  Tobacco Smoke  Environmental |
| **Cadmium** | U.S.  Canada  Germany  Japan | Brazil  China Mexico | Occupational  Food  Smoking |
| **Chromium** | U.S.  Canada  Türkiye | China  India  Kazakhstan  Russia | Occupational  Drinking Water  Other: Welding, Plating, Fabrication, and Electronics Production |
| **Dioxins** | U.S.  Australia  U.K. | China  India  Southeast Asia (e.g. Vietnam, Cambodia) | Soil*  Foodstuffs |
| **Fluoride** | U.S.  Canada  Saudi Arabia  EU Countries | Bangladesh  Brazil  Mexico  Pakistan | Water  Environmental Airborne  Other: Main exposure pathways differ between HICs (water fluoridation) and LMICs (fluoride mining) |
| **HHPs** | U.S.  Canada  Eastern Mediterranean Region Countries | China  India  Indonesia  Mexico  Russia  Ukraine | Occupational (Farming)  Environmental Food |
| **Lead** | U.S.  France  Australia  U.K | Bangladesh  India Peru  Russia  Ukraine | Occupational (battery recycling)  Paint  Environmental Food/Cookpots |
| **Mercury** | U.S.  Canada  Japan  Australia | China India  South American Countries (Peru, Brazil, Chile) | Occupational (gold mining)  Environmental Food  Environmental Airborne |
| **PAHs** | U.S.  Canada  Australia  Germany  South Africa  Türkiye | China  Ghana  India  Russia | Occupational  Environmental Airborne |
| **PCBs** | U.S.  Canada  Japan  Taiwan | Brazil  China  India Central Asian Countries | Food  Occupational Airborne |
| **PFAs** | Ubiquitous** | Ubiquitous** | Water  Consumer Goods  Occupational |
| **Phthalates** | Ubiquitous** | Ubiquitous** | Water  Food |
| **EDCs** | Ubiquitous** | Ubiquitous** | Occupational  Food |
| **Brominated Flame Retardants** | U.S.  Canada  Japan  U.K. | China  India  Indonesia  Ghana | Food  Occupational |

*** - Indicates a unanimous consensus in ranking of exposure pathways**

**** - Ubiquitous exposure makes it difficult to single out countries impacted by chemicals**
