# Supplementary material for: Structured expert judgement approach of the health impact of various chemicals and classes of chemicals
Source: PLoS One. 2024 Jun 24;19(6):e0298504. doi: 10.1371/journal.pone.0298504 (PMC11195936; doi:10.1371/journal.pone.0298504)
Supplement: S4 Table — (DOCX) [file pone.0298504.s007.docx]

**S4 Table: Example Elicitation Questions, Asbestos**

| **ASB_F1** | Premature deaths*** |
| --- | --- |
| **ASB_F2** | DALYs lost*** |
| **ASB_F3** | Percentage deaths in high-income countries |
| **ASB_F4** | Percentage deaths in low- and middle- income countries |
| 1. Countries most impacted (high-income)  2. Countries most impacted (low- and middle-income)  3. Is the current level of science regarding dose-response analysis adequate?  4. Is the current level of science regarding exposure levels adequate?  5. Please rank the major exposure pathways listed here from 1 (most severe) to 5 (least severe). Please define "other" if you rank it highly | |
