# Supplementary material for: Structured expert judgement approach of the health impact of various chemicals and classes of chemicals
Source: PLoS One. 2024 Jun 24;19(6):e0298504. doi: 10.1371/journal.pone.0298504 (PMC11195936; doi:10.1371/journal.pone.0298504)
Supplement: S5 Table — (DOCX) [file pone.0298504.s008.docx]

**S5 Table: Calibration Variables**

| **Calibration Number** | **Calibration Question** |
| --- | --- |
| **CAL01** | Global emissions of Hg resulted from artisanal and small scale gold mining in 2015 |
| **CAL02** | Median estimated number of foodborne deaths due to aflatoxins |
| **CAL03** | Estimated median global foodborne DALYs due to aflatoxins |
| **CAL04** | Estimated median foodborne illness, attributed to lead, 2015, for SEAR D sub-region |
| **CAL05** | Percentage of premature deaths due to indoor pollution in India, 2019 |
| **CAL06** | Percentage of premature deaths due to indoor pollution in Bangladesh, 2019 |
| **CAL07** | Percentage of fatalities due to unintentional pesticide poisoning in America (Caribbean, Central, North and South) |
| **CAL08** | Annual estimate of underreported of suicides in India |
| **CAL09** | Average DDT in fat in adult penguins in 2008 |
| **CAL10** | Number of deaths from occupational exposure to benzene in 2019, according to IHME |
| **CAL11** | Health workers per 10,000 in France, 2019 |
| **CAL12** | Health workers per 10,000 in Sudan, 2019 |
| **CAL13** | Mean ambient particulate matter pollution in China, 2010 |
| **CAL14** | Mean ambient particulate matter pollution in India, 2010 |
| **CAL15** | Upper limit estimated number of liver cancer cases in China in people with hepatitis B virus |
| **CAL16** | Percentage change in rate premature deaths from asthma between 2010 and 2019 |
| **CAL17** | Percentage of global premature deaths from modern pollution in 2015 |
