# Supplementary material for: Structured expert judgement approach of the health impact of various chemicals and classes of chemicals
Source: PLoS One. 2024 Jun 24;19(6):e0298504. doi: 10.1371/journal.pone.0298504 (PMC11195936; doi:10.1371/journal.pone.0298504)
Supplement: S6 Table — (DOCX) [file pone.0298504.s009.docx]

**S6 Table: Scoring results with 10 effective calibration variables:**

| ID | Statistical Accuracy | Mean rel.info | | Un-Normalize | Relative information wrt EW | |
| --- | --- | --- | --- | --- | --- | --- |
|  |  | Total | Realizations | Weight | All var. | Calibration var. |
| exprt1 | 0.001797 | 4.05 | 2.971 | 0.00534 | 3.13 | 1.505 |
| exprt2 | 0.0355 | 1.985 | 2.447 | 0.08709 | 1.659 | 1.465 |
| exprt3 | 6.36E-08 | 2.7 | 4.789 | 0 | 2.402 | 2.621 |
| exprt4 | 3.64E-07 | 3.412 | 1.859 | 0 | 2.107 | 1.355 |
| exprt5 | 1.11E-06 | 1.919 | 3.537 | 0 | 1.808 | 2.513 |
| exprt6 | 7.16E-07 | 4.651 | 2.49 | 0 | 3.016 | 1.886 |
| exprt7 | 1.52E-06 | 3.454 | 3.344 | 5.07E-06 | 2.271 | 1.509 |
| exprt8 | 0.000299 | 4.71 | 3.014 | 0.000901 | 2.995 | 1.792 |
| exprt9 | 7.16E-07 | 4.181 | 1.679 | 0 | 2.568 | 1.246 |
| PW_10 Op | 0.6933 | 1.896 | 2.278 | 1.579 | NA | NA |
| EW_10 | 0.4866 | 0.9714 | 1.137 | 0.5535 | NA | NA |
