# Supplementary material for: Structured expert judgement approach of the health impact of various chemicals and classes of chemicals
Source: PLoS One. 2024 Jun 24;19(6):e0298504. doi: 10.1371/journal.pone.0298504 (PMC11195936; doi:10.1371/journal.pone.0298504)
Supplement: S7 Table — (DOCX) [file pone.0298504.s010.docx]

**S7 Table: Robustness on Items:**

| Bayesian Updates: no  Weights: item  DM Optimisation: yes | |
| --- | --- |
| Calibration Power: 0.6000 |  |

| **Number** | **Excl. Item** | **Relative Information** | | **Calibration** | **Relative information wrt original PW** | |
| --- | --- | --- | --- | --- | --- | --- |
|  |  | **Total** | **Calibr. Vbls** |  | **Total** | **Calibr. Vbls** |
| **1** | CAL01 | 1.908 | 2.391 | 0.5608 | 0.1813 | 0.2005 |
| **2** | CAL02 | 1.90 | 2.331 | 0.5608 | 0.0696 | 0.0252 |
| **3** | CAL03 | 1.573 | 2.105 | 0.6465 | 0.1407 | 0.1419 |
| **4** | CAL04 | 1.747 | 2.308 | 0.3538 | 0.175 | 0.1135 |
| **5** | CAL05 | 1.863 | 2.138 | 0.5608 | 0.2464 | 0.1578 |
| **6** | CAL06 | 1.577 | 2.157 | 0.5608 | 0.148 | 0.1811 |
| **7** | CAL07 | 1.879 | 2.147 | 0.7315 | 0.05731 | 0.08057 |
| **8** | CAL08 | 2.395 | 2.473 | 0.7315 | 0.7751 | 0.4018 |
| **9** | CAL09 | 2.254 | 2.543 | 0.8211 | 0.5743 | 0.368 |
| **10** | CAL10 | 2.272 | 2.371 | 0.621 | 0.5742 | 0.361 |
| **11** | CAL11 | 1.888 | 2.342 | 0.5608 | 0.09043 | 0.05729 |
| **12** | CAL12 | 1.92 | 2.417 | 0.8211 | 0.1215 | 0.249 |
| **13** | CAL13 | 1.916 | 2.421 | 0.7315 | 0.05549 | 0.06258 |
| **14** | CAL14 | 1.754 | 2.366 | 0.5608 | 0.1914 | 0.1311 |
| **15** | CAL15 | 2.246 | 2.343 | 0.621 | 0.601 | 0.3156 |
| **16** | CAL16 | 1.886 | 2.182 | 0.7315 | 0.05698 | 0.07891 |
| **17** | CAL17 | 1.903 | 2.264 | 0.621 | 0.1717 | 0.3003 |
| **18** | None | 1.896 | 2.278 | 0.6933 |  |  |
