# Supplementary material for: Structured expert judgement approach of the health impact of various chemicals and classes of chemicals
Source: PLoS One. 2024 Jun 24;19(6):e0298504. doi: 10.1371/journal.pone.0298504 (PMC11195936; doi:10.1371/journal.pone.0298504)
Supplement: S8 Table — (DOCX) [file pone.0298504.s011.docx]

**S8 Table: Estimated Premature Deaths Lost as a Result of Chemicals, by Type**

| **Chemical** | **PW5%** | **PW50%** | **PW95%** | **EW5%** | **EW50%** | **EW95%** |
| --- | --- | --- | --- | --- | --- | --- |
| **ASBF1** | 4.82E+04 | 2.74E+05 | 3.46E+05 | 153.7 | 1.69E+05 | 6.80E+05 |
| **ASF1** | 21.71 | 1.36E+05 | 4.82E+05 | 21.26 | 3.07E+04 | 3.48E+05 |
| **BZF1** | 1.916 | 3,472 | 6,998 | 1.573 | 2,482 | 5.71E+04 |
| **CDF1** | 2.707 | 8,327 | 4.89E+04 | 2.613 | 1,878 | 5.35E+04 |
| **CRF1** | 4.449 | 5.90E+04 | 1.46E+05 | 1.195 | 1,732 | 1.07E+05 |
| **DF1** | 0.05017 | 47.89 | 325.7 | 0.05213 | 29.25 | 5.29E+05 |
| **FF1** | 0.05099 | 48.12 | 137.6 | 0.05547 | 29.34 | 7.82E+04 |
| **HHPF1** | 142.1 | 2.52E+05 | 4.84E+05 | 10.48 | 1.34E+05 | 2.38E+06 |
| **PBF1** | 944.9 | 1.66E+06 | 3.23E+06 | 690.7 | 4.85E+05 | 3.31E+06 |
| **HGF1** | 0.8269 | 7.98E+04 | 1.50E+05 | 0.2975 | 1,848 | 5.39E+05 |
| **PAHF1** | 8.828 | 8.99E+04 | 1.48E+05 | 2.8 | 5528 | 1.20E+05 |
| **PCBF1** | 0.2012 | 25.47 | 1989 | 0.2224 | 174.4 | 5.37E+05 |
| **PFAF1** | 0.211 | 9,460 | 5.00E+04 | 0.2345 | 741.7 | 4.44E+06 |
| **PHF1** | 0.05018 | 9.901 | 1.47E+04 | 0.05382 | 980.4 | 7.03E+05 |
| **EDCF1** | 0.05416 | 2.39E+04 | 4.52E+05 | 0.06004 | 5193 | 6.62E+05 |
| **BFRF1** | 0.05091 | 8,591 | 4.86E+02 | 0.03432 | 4313 | 3.61E+04 |
