# Supplementary material for: Structured expert judgement approach of the health impact of various chemicals and classes of chemicals
Source: PLoS One. 2024 Jun 24;19(6):e0298504. doi: 10.1371/journal.pone.0298504 (PMC11195936; doi:10.1371/journal.pone.0298504)
Supplement: S9 Table — (DOCX) [file pone.0298504.s012.docx]

**S9 Table: Estimated DALYs Lost as a Result of Chemicals, by Type**

| **Variable** | **PW5%** | **PW50%** | **PW95%** | **EW5%** | **EW50%** | **EW95%** |
| --- | --- | --- | --- | --- | --- | --- |
| **ASBF2** | 3.92E+5 | 4.229E+6 | 4.499E+6 | 1922 | 7.451E+5 | 7.253E+6 |
| **ASF2** | 378.9 | 9.867E+5 | 1.795E+6 | 316.8 | 2.816E+5 | 8.61E+7 |
| **BZF2** | 95.15 | 9.883E+4 | 1.498E+5 | 81.08 | 8.547E+4 | 2.338E+5 |
| **CDF2** | 98.79 | 1.941E+5 | 3.497E+5 | 74.02 | 60110 | 1.668E+6 |
| **CRF2** | 4.005E+4 | 2926000 | 3.977E+6 | 32.02 | 61050 | 1.668E+6 |
| **DF2** | 0.5971 | 900500 | 1.5E+6 | 0.5585 | 47970 | 2.05E+7 |
| **FF2** | 1431 | 4323000 | 2.458E+7 | 150.8 | 56710 | 2.156E+7 |
| **HHPF2** | 6241 | 9464000 | 1.498E+7 | 47.53 | 5795000 | 2.494E+7 |
| **PBF2** | 3.661E+4 | 40460000 | 6.963E+7 | 1.093E+4 | 20330000 | 8.468E+7 |
| **HGF2** | 1.821E+5 | 2953000 | 3.5E+6 | 742.4 | 1232000 | 2.223E+7 |
| **PAHF2** | 1.402E-09 | 6.149E-08 | 1.848E+6 | 3.483E-09 | 4634 | 1.671E+6 |
| **PCBF2** | 1.36E–09 | 2.884E+5 | 4.998E+5 | 2.021E-09 | 2452 | 1.587E+7 |
| **PFAF2** | 1.093E-09 | 1.834E+5 | 3.5E+6 | 2.419E-09 | 1959 | 3.727E+7 |
| **PHF2** | 1.371E-09 | 3988 | 5998 | 2.841E-09 | 3038 | 1.202E+5 |
| **EDCF2** | 3173 | 6.474E+5 | 4.994E+6 | 45.73 | 8839 | 2.991E+7 |
| **BFRF2** | 3032 | 1.992E+5 | 3.5E+5 | 1219 | 1.526E+5 | 6.663E+6 |
