# Supplementary material for: Structured expert judgement approach of the health impact of various chemicals and classes of chemicals
Source: PLoS One. 2024 Jun 24;19(6):e0298504. doi: 10.1371/journal.pone.0298504 (PMC11195936; doi:10.1371/journal.pone.0298504)
Supplement: S10 Table — (DOCX) [file pone.0298504.s013.docx]

**S10 Table: Estimated Percentage of Deaths in High Income Countries as a Result of Chemicals, by Type**

| **Variable** | **PW5%** | **PW50%** | **PW95%** | **EW5%** | **EW50%** | **EW95%** |
| --- | --- | --- | --- | --- | --- | --- |
| **ASBF3** | 0.2008 | 0.5672 | 0.6497 | 0.009023 | 0.2521 | 0.6213 |
| **ASF3** | 0.03018 | 0.1209 | 0.2123 | 0.02246 | 0.1052 | 0.2986 |
| **BZF3** | 0.2002 | 0.309 | 0.4781 | 0.003558 | 0.2689 | 0.5541 |
| **CDF3** | 0.003018 | 0.02046 | 0.02457 | 0.01012 | 0.2615 | 0.6967 |
| **CRF3** | 0.03009 | 0.0819 | 0.2956 | 0.01695 | 0.1505 | 0.5196 |
| **DF3** | 0.05014 | 0.1018 | 0.4487 | 0.01296 | 0.1291 | 0.6713 |
| **FF3** | 0.05019 | 0.1525 | 0.2811 | 0.002124 | 0.1183 | 0.4385 |
| **HHPF3** | 0.05021 | 0.1523 | 0.2725 | 0.01449 | 0.125 | 0.3517 |
| **PBF3** | 0.01011 | 0.05236 | 0.1392 | 0.01291 | 0.08065 | 0.346 |
| **HGF3** | 0.01248 | 0.1111 | 0.1785 | 0.01316 | 0.08972 | 0.46577 |
| **PAHF3** | 0.05022 | 0.1075 | 0.4656 | 0.0683 | 0.2407 | 0.5853 |
| **PCBF3** | 0.02202 | 0.387 | 0.7411 | 0.02398 | 0.1765 | 0.8068 |
| **PFAF3** | 1.427E-09 | 6.647E-08 | 0.7156 | 5.014E-09 | 0.3559 | 0.826 |
| **PHF3** | 0.2522 | 0.5332 | 0.8352 | 0.1204 | 0.4896 | 0.8331 |
| **EDCF3** | 0.2282 | 0.5006 | 0.7489 | 0.08604 | 0.2911 | 0.7579 |
| **BFRF3** | 0.25 | 0.5008 | 0.75 | 0.1116 | 0.5139 | 0.7726 |
