# Supplementary material for: Structured expert judgement approach of the health impact of various chemicals and classes of chemicals
Source: PLoS One. 2024 Jun 24;19(6):e0298504. doi: 10.1371/journal.pone.0298504 (PMC11195936; doi:10.1371/journal.pone.0298504)
Supplement: S11 Table — (DOCX) [file pone.0298504.s014.docx]

**S11 Table: Estimated Percentage of Deaths in Low- and Middle- Income Countries as a Result of Chemicals, by Type**

| **Variable** | **PW5%** | **PW50%** | **PW95%** | **EW5%** | **EW50%** | **EW95%** |
| --- | --- | --- | --- | --- | --- | --- |
| **ASBF4** | 0.3501 | 0.4297 | 0.7992 | 0.009133 | 0.6868 | 0.91 |
| **ASF4** | 0.7298 | 0.8785 | 0.9698 | 0.04681 | 0.8465 | 0.9717 |
| **BZF4** | 0.5077 | 0.6916 | 0.7998 | 0.003664 | 0.6632 | 0.9157 |
| **CDF4** | 0.7569 | 0.9796 | 0.997 | 0.2675 | 0.7384 | 0.9898 |
| **CRF4** | 0.6051 | 0.9173 | 0.9699 | 0.283 | 0.7977 | 0.9828 |
| **DF4** | 0.5612 | 0.8985 | 0.9499 | 0.1003 | 0.6477 | 0.9787 |
| **FF4** | 0.7074 | 0.8475 | 0.9499 | 0.03047 | 0.8375 | 0.9975 |
| **HHPF4** | 0.7104 | 0.8476 | 0.9498 | 0.08994 | 0.8209 | 0.9829 |
| **PBF4** | 0.706 | 0.8565 | 0.95 | 0.01048 | 0.8781 | 0.9857 |
| **HGF4** | 0.8058 | 0.8818 | 0.9699 | 0.3584 | 0.7589 | 0.9212 |
| **PAHF4** | 0.565 | 0.8445 | 0.9496 | 0.35844 | 0.7589 | 0.9212 |
| **PCBF4** | 0.2546 | 0.5634 | 0.9702 | 0.045 | 0.4651 | 0.9647 |
| **PFAF4** | 1.427E-09 | 6.647E-08 | 0.7156 | 5.089E-09 | 0.3649 | 0.8768 |
| **PHF4** | 0.2124 | 0.4268 | 0.745 | 0.2214 | 0.5271 | 0.8807 |
| **EDCF4** | 0.2422 | 0.4987 | 0.7578 | 0.1222 | 0.5888 | 0.8928 |
| **BFRF4** | 0.25 | 0.4992 | 0.75 | 0.2274 | 0.4861 | 0.8884 |
